# Storage and Packaging Effects on the Protein Oxidative Stability, Functional and Digestion Characteristics of Yak Rumen Smooth Muscle

**DOI:** 10.3390/foods11142099

**Published:** 2022-07-14

**Authors:** Zhuo Wang, Xiaobo Liu, Theodora Ojangba, Li Zhang, Qunli Yu, Ling Han

**Affiliations:** 1 College of Food Science and Engineering, Gansu Agricultural University, Lanzhou 730070, China; wangzhuo1030@163.com (Z.W.); theodora@st.gsau.edu.cn (T.O.); yuql@gsau.edu.cn (Q.Y.); hanl@gsau.edu.cn (L.H.); 2 Gansu Research Institute of Light Industry Co., Ltd., Lanzhou 730070, China; liuxb@st.gsau.edu.cn

**Keywords:** rumen, smooth muscle protein, oxidative stability, functional properties, in vitro digestion

## Abstract

The objective of this study was to investigate the effects on protein oxidative stability, functional and digestion characteristics of yak rumen smooth muscle with overwrap packaging using oxygen-permeable film (OWP) and vacuum packaging bag (VP) during storage (0, 7, 14, 28, 42, 56, 84, 168 and 364 days) at −18 °C. The results show that yak rumen smooth muscle was oxidized with frozen storage through the formation of protein carbonyls and disulfide bonds, the loss of total sulfhydryl. The emulsifying activity of yak rumen smooth muscle protein (SMP) under VP began to perform a higher level than that under OWP after 14 days, and the foaming capacity under VP showed the highest level on the 28th day of 111.23%. The turbidity under VP reached the minimum 0.356 on the 28th day as well, followed by significantly increasing on the 56th day compared with OWP. The digestibility of yak rumen SMP under both OWP and VP reached the maximum on the 28th day of frozen storage. Moreover, yak rumen under VP at 28–56 days of frozen storage had good functional properties and high digestibility of SMP, which showed better edible value.

## 1. Introduction

Smooth muscle is a sort of muscle tissue that widely exists in the gastric organs, intestines and other animals’ internal organs [1]. With the gradual increase in livestock slaughtering, the amount of gastrointestinal by-products full of smooth muscle has increased as well [2]. These edible by-products can be further utilized by humans as food because of their distinctive taste and high nutritional value. Rumen-based foods are still popular in oriental cuisine and traditional meat snacks in Asia; they can be cooked into a cold appetizer and soup in China and Korea, as well as rolls in India [3,4]. Rumen is also extensively used as casings and fillings of traditional semi-dry fermented sausages in Spain [5]. There are three main mechanisms for meat and meat product spoilage during storage: microbial spoilage, protein/lipid oxidation and autolysis enzymatic spoilage [6]. As a good resource of protein and product development, rumen meat contains 24.5% protein, 8.4% fat and 66.6% water [7]. However, high protein and water content largely lead to the poor preservability caused by oxidation and microorganisms; they also cause rumen meat to be limited by many exploitations in commercial application [8,9].

Freezing and vacuum packaging are commonly used storage methods for perishable items such as meat and meat products [10,11]. Low temperatures resist microbial spoilage and reduce the color, texture and odor defect caused by microbiology [12,13], such as the “blue pork” or “blue beef” caused by blue pigment and the slime formation on meat surface as a result of *L. sakei* strains [14]. However, the formation of ice crystals during storage can lead to sustained physical disruption to the cell structure, as well as chemical reactions including protein and lipid oxidation [15,16,17]. Oxygen-permeable packaging has film with holes, pores, or perforations that allow oxygen to diffuse from the atmosphere; this is a commonly used packaging type for the retail display of raw chilled meat, and most families use it for short-term meat storage. Compared with OWP, vacuum packaging offers a longer shelf life of meat because of its strong suppressive effects on low oxygen oxidation and microbe; however, this minimal oxidation reaction influences the mechanical, sensory and nutritional nature of meat and meat products as well [18,19]. Protein oxidation develops toxic compounds and odor and negatively influences the texture and water-holding capacity by decomposition and denaturation of the meat protein. The accumulation of protein oxidation products during storage means that the potential further oxidation of dietary proteins during digestion may worsen the potential negative influence of protein oxidation on nutritional and health issues.

Smooth muscle cells are composed of thick, thin and intermediate filaments. The function of smooth muscle cells’ thick and thin filaments is identical to that of myosin and actin skeletal muscle cells, and the contraction rules of both are also therefore similar [17,18]. The change law of skeletal muscle’ protein properties under different packaging methods during frozen storage have been studied a lot previously [20,21,22]. Considering the similarities in the structure of both smooth and skeletal muscle, whether smooth muscle protein (SMP) properties follow similar trends is unknown. Therefore, since limited information is available about the effects on quality changes of yak rumen smooth muscle with different packaging during frozen storage. The purpose of this paper was to study the effects of frozen storage and packaging method on the oxidative stability, functional and digestion characteristics of yak rumen SMP.

## 2. Materials and Methods

### 2.1. Animals and Sample Collection

The smooth muscle in this paper was obtained from the rumen of the yak (Bos grunniens). Nine yak bulls (mean weight: 240 kg; mean age: 38 months) were obtained from a commercial abattoir in Gannan, Gansu Province, China. The rumen was obtained immediately after humanely slaughtering in compliance with the Operating procedure of livestock and poultry slaughtering—Cattle of the National Standards of P.R. China [23]. The smooth muscles have been sliced into cubes (length × width of 50 × 50 mm, natural thickness) after all visible fat and mucosa were removed. In total, 12 pieces of samples with relatively uniform thickness were cut from each yak rumen. Subsequently, 108 pieces of rumen samples were randomly subdivided into two fractions for OWP and VP packaging systems. In total, 54 pieces were packaged with oxygen-permeable film bag (OWP) and the rest of the pieces were packaged with vacuum packaging film bag (VP). After being packaged, they were transferred to the laboratory in ice packs within 2 h. All the samples were then stored for 0, 7, 14, 28, 42, 56, 84, 168 and 364 days at −18 °C. Afterwards, 6 pieces were randomly picked out from each packaging treatment for determination and analysis at the end of each designated storage times.

### 2.2. Packaging Treatments

Sample for OWP was packaged into a poly ethylene (PE) bag with a thickness of 150 μm. The rate of transmission of oxygen was 81.86 cm^3^/m^2^/24 h/bar at 23 °C and 0% relative humidity, and the rate of transmission of water vapor was 3.28 g/m^2^/24 h at 38 °C and 100% relative humidity.

A vacuum machine (DZ-450A, Dajiang Vacuum Packaging Machinery Co., Ltd., Wenzhou, China) was used for VP. The VP packaging bag was a PE bag with a thickness of 180 μm, its oxygen transmission rate was 23.27 cm^3^/m^2^/24 h/bar at 23 °C and 0% relative humidity, and the rate of transmission of water vapor was 2.94 g/m^2^/24 h at 38 °C and 100% relative humidity.

### 2.3. Extraction of Yak Rumen Smooth Muscle Protein

The SMP was prepared using the Young and Lawrie [24] process with small modifications. A total of 10 g of the muscle separated from the surface of the black membrane was homogenized (SRH 60–70, Shanghai shen deer homogenizer Co., Ltd., Shanghai, China) in the ice bath (10,000 rpm, 20 s, 3 times) with 40 mL 0.6 mol/mL NaCl (pH 7.0). The homogenate was stirred continuously for 2 h at 20 °C, then centrifuged at 1000× *g* at room temperature for 15 min, leaving the supernatant with filtration. The concentration of protein was determined by the biuret procedure.

### 2.4. Oxidative Stability

#### 2.4.1. Determination of Carbonyl Content

Protein carbonyls were assayed as hydrazone derivatives by reacting proteins with 2,4-dinitrophenylhydrazine (DNPH) as Levine et al. [25] described, with some modifications. A total of 50 µL sample protein solution aliquot was reacted with 2 mL of 10 mM DNPH in 2 M HCl for 1 h at room temperature; control was the same as above besides DNPH. Add 2 mL 20% (*w/v*) trichloroacetic acid (10,000 rpm, 4 °C, 5 min) and discard the supernatant. Wash the precipitate with 2 mL ethyl acetate: Ethanol (1:1) for 3 times until the reagent volatilizes completely. Add 3 mL 6 M guanidine hydrochloride (containing 20 mmol/L sodium phosphate, pH 6.5) solution, the precipitate was dissolved in a 37 °C water bath for 30 min and then centrifuged at 10,000× *g* for 5 min (4 °C). The absorbance of the supernatant was measured at 370 nm and the carbonyl content was calculated with a molar extinction coefficient of 22,000 M^−1^·cm^−1^.

#### 2.4.2. Determination of Total Sulfhydryl Group

Total sulfhydryl content was carried out according to Li et al. [26]. The concentration of sample protein solution was adjusted to 2 mg/mL by 25 mmol/L sodium phosphate buffer (pH 6.25). Aliquots of 0.5 mL of protein suspension was dissolved in 2.0 mL urea- sodium dodecyl sulfate (SDS) solution (8.0 M urea, 3% SDS, 0.1 M phosphate, pH 7.4) and 0.5 mL 5,5′-Dithiobis-2-nitrobenzoic acid (DTNB) (10 mmol/L in 0.1 mol/L sodium phosphate buffer, pH 7.4). The absorbance of the supernatant was estimated at 412 nm after the reaction at room temperature for 15 min. Sulfhydryl content was calculated from an extinction coefficient of 13,600 M^−1^·cm^−1^.

#### 2.4.3. Determination of Disulfide Bond

The disulfide bond was measured according to Thannhauser et al. [27] with minor modifications. In the buffer solution of 25 mmol/L Sodium Phosphate (pH 6.25), the smooth muscle protein was modified to 5 mg/mL. A total of 100 μL of protein solution was pipetted to 3 mL of NTSB assay solution. The reaction mixture was incubated in the dark for 25 min. The absorbance at 412 nm was recorded and the blank was distilled water.

#### 2.4.4. Determination of Surface Hydrophobicity

SMP’s surface hydrophobicity was measured using the techniques of Chelh et al. [28]. SMP was balanced to 1 mg/mL by 20 mM of sodium phosphate buffer solution (pH 6.0). In total, 80 μL of 1 mg/mL bromophenol blue was added to 2 mL protein solution and blended properly. Samples and control were held under agitation at room temperature for 10 min, then centrifuged at 4000× *g* for 15 min (4 °C). The absorbance of the supernatant (diluted 10-fold) was estimated at 595 nm.

$$\mathrm{Bromophenol}\;\mathrm{blue}\;(\mu g)=80\;\mu g\times \frac{A_{Blank}-A_{Sample}}{A_{Blank}}$$

### 2.5. Functional Characteristics

#### 2.5.1. Determination of Solubility

The solubility of smooth muscle proteins, as defined by Li et al. [29] and Joo et al. [30], was calculated with minor adjustment. A total of 1 g of sarcoplasmic protein was obtained as a supernatant in 30 mM sodium phosphate buffer at pH 7.4 with 0.02% NaN_3_, inserting 20 mL of ice cold 1.1 M potassium iodide in 0.1 M phosphate buffer (pH 7.2), shaking for 20 s, 10,000 rpm for 3 times, and then leaving on a shaker at 4 °C for 12 h. Samples were centrifuged at 1500× *g* for 20 min (4 °C) and the concentration of protein in the supernatants was measured by the Biuret process.

$$\mathrm{Solubility}\left(\mathrm{mg}/g\right)=\frac{\mathrm{Protein}\;\mathrm{content}\;\mathrm{of}\;\mathrm{supernate}}{\mathrm{Total}\;\mathrm{protein}\;\mathrm{content}\;\mathrm{of}\;\mathrm{sample}}$$

#### 2.5.2. Determination of Emulsify Properties

The emulsify properties were measured according to Li et al. [31] with minor changes. In total, 10 mL of corn oil and 30 mL of 1 mg/mL SMP solution in 0.1 M phosphate buffer (pH 7.0) were shaken together then homogenized at 10,000 rpm for 1 min at room temperature. A total of 200 μL of emulsions was taken from the bottom of the container after being placed undisturbed for 10 min, and dispersed into 10 mL of 0.1% SDS solution. The absorbance was recorded at 500 nm with 0.1% SDS as a blank control. Emulsifying activity index (EAI) and emulsion stability (ES) were defined as:

$$\mathrm{EAI}\left(m^{2}/g\right)=\frac{2\times 2.303}{C\times \left(1-\varphi \right)\times 10^{4}}\times A_{0}\times dilution$$

$$\mathrm{ES}\;(\%)=\frac{A_{10}}{A_{0}}\times 10$$

where *A*_0_ and *A*_10_ represent the absorption value of the emulsion at 0 and 10 min, respectively. C represents the protein concentration (g/mL) before emulsification (C = 0.001), φ represents the oil volume fraction (*v/v*) of the emulsion (φ = 0.20).

#### 2.5.3. Determination of Foaming Properties

Foaming properties were carried out by using the method of Plancken et al. [32]. In total, 50 mL of SMP solution (1 mg/mL, pH 7.0) foaming solution was added into a 100 mL graduated cylinder, and whipped with a rotating anchor at 15,000 rpm for 2 min at room temperature. The height *V*_0_ of foam was recorded after the end of whipping, and *V*_30_ after 30 min. The foaming capacity (FC) and foaming stability (FS) were calculated according to the following formulas, respectively:

$$\mathrm{FC}\;(\%)=\frac{V_{0}-50}{100}\times 100\%$$

$$\mathrm{FS}\;(\%)=\frac{V_{30}-50}{V_{0}-50}\times 100\%$$

where *V*_0_ and *V*_30_ represent the height of foam after the end of whipping at 0 and 30 min, respectively.

#### 2.5.4. Determination of Turbidity

The turbidity was measured according to the method of Benjakul et al. [33], and 5 mL protein solution, which was adjusted to 1 mg/mL, was added into a 10 mL centrifuge tube, heated in water bath at 80 °C for 30 min and then cooled to room temperature. Protein aggregation can be monitored continuously by measuring the absorbance at 660 nm.

### 2.6. In Vitro Digestion

The samples were precooked in an 85 °C water bath for 12 min before being digested. The SMP digestibility was assessed as previously described by Escudero et al. [34] and Wen et al. [35] with slight modifications.

Samples (3.0 g, each) were homogenized in 12 mL of PBS solution (33 mM glycine buffer) for 3 × 20 s at 10,000 rpm. The homogenate was adjusted to pH 1.8 ± 0.1 with 1 mol/L HCl, and pepsin (≥250 units/mg) was added 0.01 g at the sample. The mixture was incubated at 37 °C for 1.5 h with continuous shaking (VWR International, LLC., West Chester, PA, USA), and then the enzyme was inactivated by adjusting the pH to 8.0 with 1 mol/L NaOH. After gastric digestion, intestinal digestion was followed, 0.01 g trypsin (>250 units/mg) and 0.001 g of chymotrypsin (>1200 units/mg) were added at sample, and the mixture was incubated at 37 °C for 2 h with continuous shaking. After that, enzyme activity was inactivated by heating at 100 °C for 5 min. Each digestion process was deproteinized by adding three volumes of ethanol and then centrifuged at 10,000× *g* for 20 min at 4 °C. The supernatant was discarded and precipitate was used for the next test.

Three portions of sample weighing 3 g were taken from each piece. One portion was only treated with pepsin; the reaction stopped when digestion was finished, which would be used to calculate the gastric digestibility. The second portion was treated with trypsin and chymosin to get the intestinal digestibility. Additionally, the last portion was treated with pepsin, followed by trypsin and chymosin. The digestion procedures were the same as above. After digestion, the digest was centrifuged at 1000× *g* for 20 min at 4 °C and the supernatant was discarded. The precipitate was dried until the weight change of insoluble protein was achieved as shown in the following equation:

$$\mathrm{DT}\left(\%\right)=\frac{W_{0}-W_{1}}{W_{0}}\times 100\%$$

where DT is the digestibility, *W*_1_ is the weight of dried insoluble protein, and *W*_0_ is the total weight of sample before digestion.

### 2.7. Statistical Analysis

SPSS software (SPSS, Version 20.0, IBM Co., Somers, NY, USA) was used for statistical analysis following General Linear Models (GLM) procedure. The model included time of storage and packaging treatments (as fixed effects) and their interaction (storage time × packaging). The dependent variables were carbonyl, total sulfhydryl, disulfide bond, and surface hydrophobicity; solubility and emulsifying properties; foaming properties, turbidity and in vitro digestibility. Two-way analysis of variance (ANOVA) was used to analyze the interactions between fixed factors at a significance level of 0.05. After significant interaction (*p* < 0.05) between the storage and packaging treatment was noted on the variables, changes of oxidative stability, functional and digestion properties among packaging treatments for each storage day and one packaging treatment over various days were calculated by one-way analysis of variance. Duncan’s multiple range tests were used to assess differences between mean values when *p* < 0.05. The principal component analysis (PCA) was carried out using Origin software (version 2021).

## 3. Results

### 3.1. Oxidative Stability

#### 3.1.1. Carbonyl

**Figure 1:**
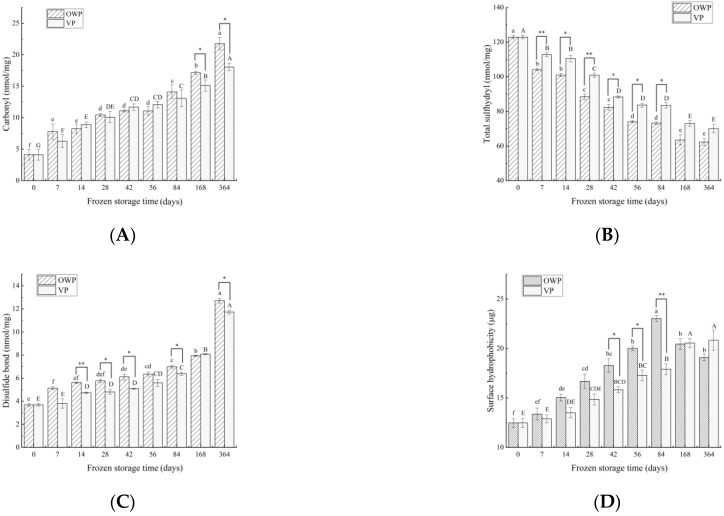
Changes in carbonyl (**A**), total sulfhydryl (**B**), disulfide bond content (**C**) and surface hydrophobicity (**D**) of yak rumen SMP during frozen storage. Different superscript letters in the figure indicate significant difference in storage time (*p* < 0.05); lowercase letters are used for OWP and uppercase letters for VP. “*” indicates that the different packaging is significant (*p* < 0.05), and “**” indicates that the different packaging is extremely significant (*p* < 0.01).

Carbonyl content of SMP generally increased during frozen storage (Figure 1A). Carbonyl content of yak rumen SMP under VP showed (*p* < 0.05) a lower level comparing with OWP at most of the storage time. Changes in both packaging methods can be similarly divided into three stages: From 0 to 14 day, the amount of carbonyls in VP and OWP increased significantly (*p* < 0.05) from 4.13 nmol/mg to 8.24 and 8.89 nmol/mg, respectively; then, they showed a non-significant trend till 84 day; during the last period from 84 to 364 day, carbonyls increased significantly again as beginning (*p* < 0.05).

#### 3.1.2. Total Sulfhydryl

Contrary to carbonyl, the total sulfhydryl content of SMP in both packaging methods decreased (Figure 1B), while in VP it always showed a higher value to it in OWP (*p* < 0.05). However, the level of total sulfhydryl tended to become stable as storage time prolonged. The content of total sulfhydryl in OWP dropped significantly (*p* < 0.05), by 50%, from its initial value (122.92 nmol/mg) to 62.29 nmol/mg at 364 day, and 70.10 nmol/mg at ^36^4th day for VP.

#### 3.1.3. Disulfide Bond

The disulfide bond (SS) content showed a continuous upward trend, corresponding to a gradual decrease in the SH content of yak rumen SMP being seen during storage and increasing significantly (*p* < 0.05) after 168 days (Figure 1C). Basically, the SS content with OWP remained significantly higher than it did with VP (*p* < 0.05).

#### 3.1.4. Surface Hydrophobicity

The surface hydrophobicity of SMP under VP showed a steady increase during storage, while with OWP it reached the maximum at 84 days, followed by a significant decrease (*p* < 0.05) from Figure 1D.

### 3.2. Functional Characteristics

#### 3.2.1. Solubility

**Figure 2:**
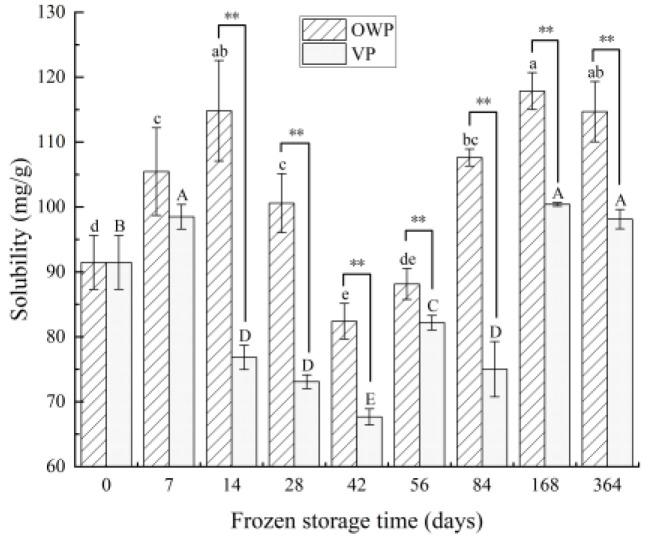
Changes in solubility of yak rumen SMP during frozen storage. Different superscript letters in the figure indicate significant difference in storage time (*p* < 0.05); lowercase letters are for OWP and uppercase letters for VP. “*” indicates that the different packaging is significant (*p* < 0.05), and “**” indicates that the different packaging is extremely significant (*p* < 0.01).

The solubility of yak rumen SMP can be observed in Figure 2, and both two-packaging systems showed roughly the same change trends. The solubility initially increased and then went down to a minimum, followed by a rising trend, which then reduced again during subsequent storage (*p* < 0.05). Solubility content peaked under both OWP and VSP (*p* < 0.05) at 168 days, 117.84 and 100.39 mg/g, respectively. Comparing the solubility value of day 168 VS 14 with OWP, and day 168 VS 7 with VP, there was no significant difference between their two peaks (*p* > 0.05).

#### 3.2.2. Emulsifying Properties

**Figure 3:**
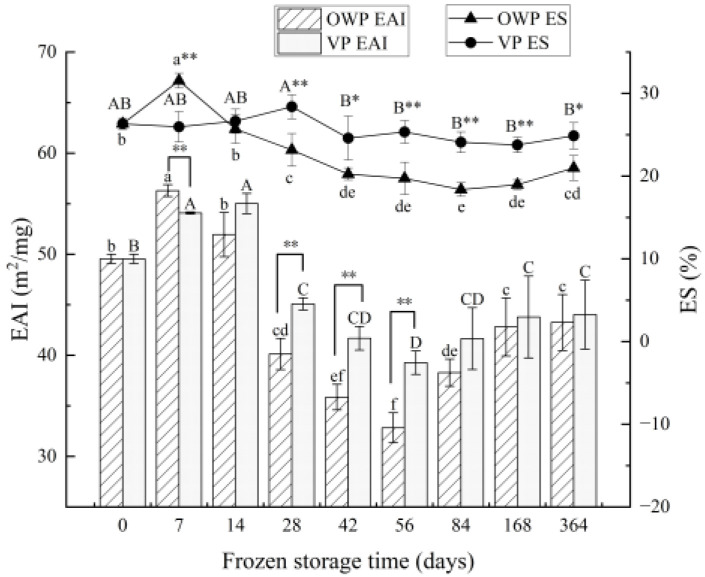
Changes in emulsifying activity index (EAI) and emulsifying stability (ES) of yak rumen SMP during frozen storage. Different superscript letters in the figure indicate significant difference in storage time (*p* < 0.05); lowercase letters are for OWP and uppercase letters for VP. “*” indicates that the different packaging is significant (*p* < 0.05), “**” indicates that the different packaging is extremely significant (*p* < 0.01).

Changes in the EAI and ES of yak rumen SMP during frozen storage are shown in Figure 3. The variation trends of EAI under different packaging were identical: it increased significantly at first (*p* < 0.05), until it reached the peaks at 7 days and 14 days for OWP and VP, respectively; then, it decreased from their maximums to the lowest level at day 56, while the EAI under VP remained significantly higher than it did under OWP; ultimately, it increased. This illustrates the hypoxic environment significantly inhibit the decrease in EAI. Similar to the pattern of EAI, ES under VP reached its maximum at 84 days, later than it did under OWP; then, it maintained an extremely significant higher level (*p* < 0.01) among the decrease before levelling off.

#### 3.2.3. Foaming Properties

**Figure 4:**
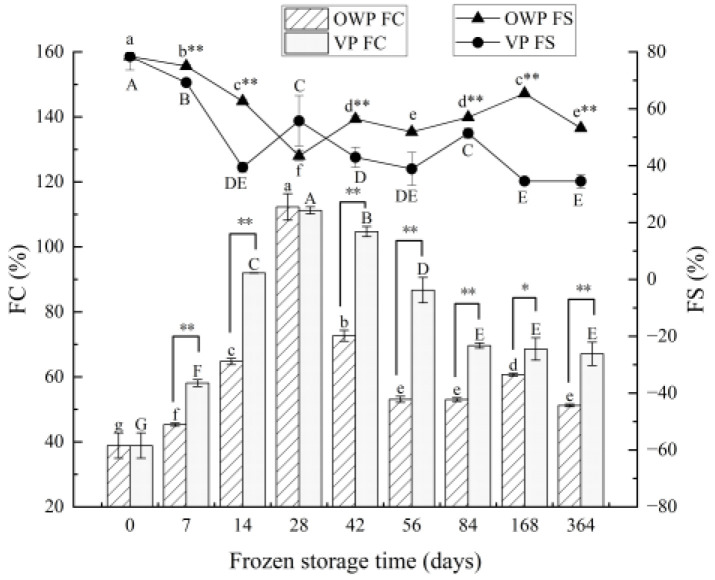
Changes in foaming capacity (FC) and foaming stability (FS) of yak rumen SMP during frozen storage. Different superscript letters in the figure indicate significant difference in storage time (*p* < 0.05); lowercase letters are for OWP and uppercase letters for VP. “*” indicates that the different packaging is significant (*p* < 0.05), and “**” indicates that the different packaging is extremely significant (*p* < 0.01).

Changes in the FC and FS of yak rumen SMP during frozen storage were shown in Figure 4. The FC value of both two-packaging systems showed the same changing trend, rising significantly at first (*p* < 0.05), then reducing significantly (*p* < 0.05). The peak values were reached at 28 days, 112.28% for OWP and 111.23% for VP. As storage time prolonged, the foaming stability of SMP decreased gradually. FS value under OWP reduced significantly from 0 to 28 day (*p* < 0.05), then it tended to be stable; FS under VP reached the lowest value at 14 days, and it achieved a stable state with the storage time keeping on going up until 168 days, when there was no significant difference (*p* > 0.05).

#### 3.2.4. Turbidity

**Figure 5:**
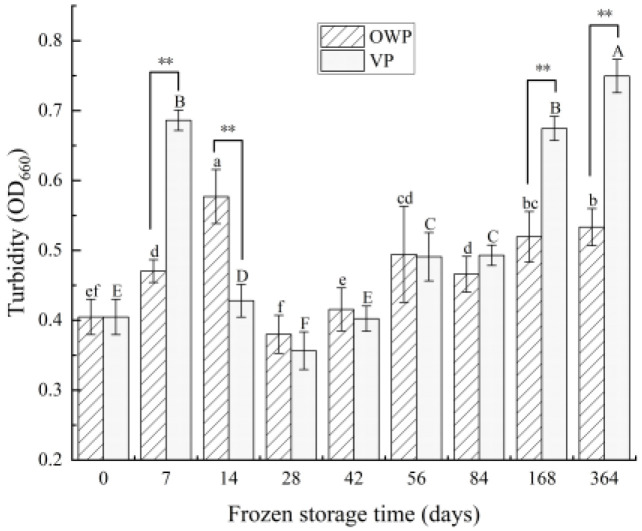
Changes in turbidity of yak rumen SMP during frozen storage. Different superscript letters in the figure indicate significant difference in storage time (*p* < 0.05), lowercase letters are for OWP and uppercase letters for VP. “**” indicates that the difference packaging is extremely significant (*p* < 0.01).

Figure 5 shows that the turbidity level of SMP increased at first, after a reducing trend, then it increased again. Turbidity value under OWP and VP increased significantly on the 14th day and the 7th day by 42.55% and 69.53%, respectively (*p* < 0.05). Then, both of them achieved the minimum value on the 28th day, 0.379 for film and 0.356 for vacuum. After 168 days, turbidity with OWP showed a non-significant increase (*p* > 0.05). However, turbidity under VP still increased significantly from 28 days to 364 days (*p* < 0.05).

### 3.3. In Vitro Digestibility

Table 1 shows the digestibility of yak rumen SMP in OWP and VP systems. The digestibility of SMP under VP still showed a higher (*p* < 0.05) level than it was under OWP. Digestibility with both OWP and VP showed a significant increase, which was later reduced (*p* < 0.05). With the frozen storage prolonged, gastric digestibility, intestine digestibility and total digestibility all achieved the maximum value at 28 days and they were significantly higher than the other three at the other time points (*p* < 0.05). This indicates that freezing time significantly affects the digestibility of protein.

### 3.4. Principal Component Analysis (PCA)

**Figure 6:**
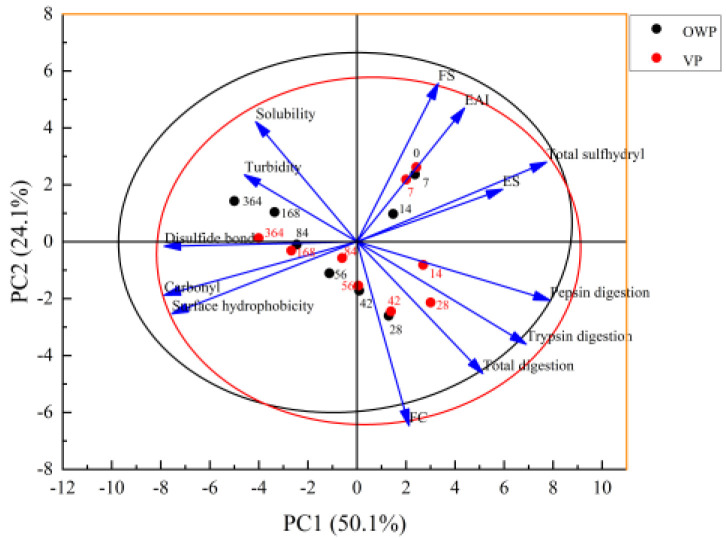
Loading plot of the first two principal components of yak rumen smooth muscle protein properties as affected by OWP and VP packaging treatments.

PCA performed yak rumen smooth muscle protein properties as affected by OWP and VP packaging, explaining 74.2% of the total variance (Figure 6). Principal component 1 (PC1) accounted for 50.1% of the total variance and was associated to total sulfhydryl, pepsin digestion, trypsin digestion and in the opposite direction disulfide bond, carbonyl and surface hydrophobicity. It can be considered as the reflection of the oxidation and digestion of SMP. On the other hand, PC2 was mainly related to protein functional parameters, such as FS, EAI, solubility, turbidity and FC. Compared with OWP packaging, VP packaging moved sequentially from the negative quadrant of PC1 to its positive quadrant. Parameters in both PC1 and PC2 negative quadrants, such as carbonyl, disulfide bond and surface hydrophobicity, were higher in OWP compared with them in VP, and with the shift to rightward, indexes such as total sulfhydryl were lower in OWP. However, the digestion characters and FC were loaded in the negative quadrant of PC2, which was in line with the results of the first increasing and then reducing of these indicators (Table 1, Figure 4). The red midpoint of day 56 was in the line of the PC1 original point, the ranges made of by day 56 and day 28 also being consistent with the results of the highest digestion value.

## 4. Discussion

Protein oxidation resulted in its structure change, and it is well known that changes in protein structures are a major determining factor in their functional attributes. Furthermore, affecting the structural and functional properties of the proteins, such as the adjusting of the protein oxidation degree, can induce denaturation and affect the unfolding and refolding of the proteins, thereby affecting both the diffusion of gastrointestinal proteases deep into the protein matrix as well as their accessibility to cleavage sites.

The oxidative stability of yak rumen SMP during freezing storage was similar to that of skeletal muscle from previous studies. With prolonged storage time, carbonyl and disulfide bonds increased and sulfhydryl groups decreased; SMP under VP showed lower oxidation levels compared with OWP. Consistent with the findings of Yang et al. [36] in beef that anaerobic packaging can effectively induce the increase in carbonyl content during storage. The significant increase in carbonyl after 84 days was similar to the result of what has been reported in chicken leg meat after 3 months frozen storage at −7 °C by Soyer et al. [37], which could be explained by the fact that long-term frozen storage would lead to the formation of large intra and extracellular ice crystals, destroy the cell structure and then result in the release of oxidative enzymes and other pro-oxidants from various ruptured cellular organelles. According to Sante-Lhoutellier [38] and his colleagues’ study of pig longissimus dorsi, when the carbonyl content reached a maximum of 16.3 nmol/mg protein, the consequences have mainly been identified as deterioration of tenderness and juiciness. Furthermore, oxidative modifications of proteins can lead to loss of essential amino acids and decreased digestibility affecting ultimately the nutritional quality of muscle foods. Lund et al. [39] also found the sulfhydryl content increasing in porcine longissimus dorsi, and skin packaging showed that the content of the free thiol is significantly increased compared to the high oxygen atmosphere packing during chill storage, which indicated that the formation of total surfhydryl could be inhibited under a low oxygen environment. This is because protein oxidation stimulated the conversion from SH to SS with time prolonged [40]. The total sulfhydryl content showed a non-significant difference from 56 days to 84 days in both two-packaging systems, which was in agreement with the result of Li et al. [25]. In this research about the protein of pork sausage, the SH content stopped falling from 55 to 90 day. This may be due to the fact that thiol groups embedded within the hydrophobic groups were easily oxidized and exposed, resulting in the non-decreasing of the total sulfhydryl content [41]. This sharp rise in S-S content after 168 days may be attributed to the complex oxidation of thiol in meat, as a variety of oxidation products may be produced, such as sulphenic acid, sulphonic acid, sulphonic acid and thiosulfinate. Other thiol oxidation products other than disulfides can be produced in meat during storage. Foaming ability is dependent [42] and can be used to detect protein conformation changes and assess protein denaturation [43]. The general upward trends of surface hydrophobicity during storage were similar to the finding by Li et al. [44] in the myofibril of common carp that protein oxidation could lead to the unfold of myofibrillar proteins and the exposure of nonpolar amino acids resulting in the increase in surface hydrophobicity. However, the fluctuation changes of it under OWP may be due to the fact that hydrophobic groups are gradually exposed because of the contact between SMPs and oxygen under oxygen-permeable packaging, but SMPs may gather in large numbers under strong hydrophobic interactions, thereby leading to the reduction in surface hydrophobicity [45].

The functional properties of proteins depend on their molecular composition and structural characteristics, and they are influenced by the external environment [46]. Although the contractile mechanisms of smooth muscle cells are similar to those of striated muscle cells, the significant difference between the different types of muscle is the difference in the molar ratio of myosin to actin. Using electron microscopy and electrophoresis tests to analyze contractile proteins in smooth muscle, it was found that the ratio of myosin to actin was 1:16 in bovine rumen smooth muscle and 1:15 in vascular smooth muscle [47,48], whereas the ratio in skeletal muscle was only 1:6 [49]. It can be seen that smooth muscle and skeletal muscle have very different protein compositions, and this difference results not only in differences in their contractile mechanisms and exercise functions, but also in differences in functional properties between proteins. Yak rumen SMP contains a high amount of connective tissue proteins, which can be well dispersed and solubilized under certain conditions. The decrease in solubility at an early period was similar with the results of Yang et al. [36] about beef during early postmortem. The creation of intracellular or intercellular ice crystals during freezing resulted in the destruction and weakening of the forces that maintain the native protein structure with the concomitant unfolding of the molecules. It was possible to cross-link unfolded myofibrillar protein molecules with other proteins through secondary interactions and disulfide bridges to form insoluble protein–protein aggregates [50]. With storage time prolonged, the structures between proteins could be destroyed by the surface charges on protein, leading to the change of solubility. The difference in emulsification capacity between smooth muscle and skeletal muscle may be attributed to differences in contractile protein structure, and it has been suggested that the difference is mainly concentrated in myosin. In this work, the increase after 84 days was because the less percentage of myosin in SMP demonstrated the helpful effect on emulsifying properties of SMP, which was in agreement with the conclusion of Xia et al. [51,52,53] that the emulsification of yak rumen SMP is higher than that of porcine longissimus muscle protein. Foaming ability is dependent upon the rate of protein denaturation, which reveals the quality of proteins. Du et al. [54] found that the foaming ability of proteins increased with the solubility of small molecules, and the order of influence on foaming properties was actin, tropomyosin, promyosin, and serum albumin, as well as some other small molecule components. Similar with the first increase then decrease change trend of SMP solubility, its foaming capacity also decreased after an increase. According to Xia et al. [51], the increased turbidity of the myofibrillar protein solutions suggested the development of protein aggregates that were wide enough to induce light scattering. Peng et al. [55] also revealed that the loss of the sulfhydryl groups may contribute to the forming of disulfide bonds either within polypeptides or between polypeptides that cause protein aggregation.

There is no doubt that packaging methods and the oxidative stability of meat products have a direct relationship. Meanwhile, controversy exists around the effects of protein oxidation modifications on the susceptibility of meat proteins to proteolytic enzymes and, hence, on their digestibility. That means there is a certain relation between the packaging methods and the protein digestibility of meat and meat products, which needs to be further studied and established. However, it seems reasonable to consider that these properties are dependent on the specific conditions under which the proteins are oxidatively modified, as well as the conditions under which the proteins are digested. Consistent with the findings of a previous study, myofibrillar proteins of porcine undergo a chemical oxidation mechanism prior to the hydrolysis by digestive proteases, and there is a high negative correlation between carbonyl levels and protease activity [56]. The digestibility of yak rumen SMP with VP showed a higher level than it did with OWP; this explains why anaerobic packaging would help rumen with a higher nutritive value. However, different to the result of Zhou et al. [57] on pork belly, the digestibility of yak rumen SMP during frozen storage under both two-packaging systems showed a decline followed by an increase, while it still decreased for pork belly during the whole 120 days.

Previous studies have shown that the chemical oxidation of myosin may affect the sensitivity of myosin to protein hydrolysis by digestive tract enzymes. Loss of digestibility is associated with myosin oxidative parameters, such as hydrophobic changes, aggregation and carbonylation [58]. Li et al. [59] indicated that while oxidatively modified proteins were proteolytic, oxidatively interlinked myosin was proteolytic resistant. This changing trend may be due to the different molar ratio of myosin to actin in SMP.

## 5. Conclusions

Storage time and packaging methods had significant effects on protein oxidative stability, functional and digestion characteristics of yak rumen SMP. In this work, VP could effectively delay the oxidation, increase the emulsifying and foaming properties, and improve the digestibility of yak rumen SMP during the frozen process compared with OWP. The 28–56-day frozen storage period yak rumen under VP with good functional properties and high digestibility of SMP can be used as a good processed and digestible source of dietary nutrition for humans. This would provide a guide for people to choose the frozen rumen for family cooking and for the food processing company to make much better use of yak rumen, since the functional and digestion properties were affected by the protein composition. This would be an obvious area to continue to study in order to gain a better understanding of the protein properties in relation to its composition in meat.

## Figures and Tables

**Table 1:** The in vitro digestibility of yak rumen SMP during frozen storage.

Digestion Pattern	Packaging	Storage Time (Days)									*p* Value
		0	7	14	28	42	56	84	168	364	
**Pepsin digestion**	OWP	55.76 ± 0.77 ^bc^	56.90 ± 0.18 ^b^	57.89 ± 0.42 ^ab^	59.49 ± 0.07 ^a^	56.29 ± 0.28 ^b^	55.74 ± 0.60 ^bc^	53.65 ± 0.84 ^c^	50.06 ± 0.40 ^d^	45.49 ± 0.61 ^e^	<0.01
	VP	55.76 ± 0.77 ^b^	57.58 ± 0.36 ^ab^	59.21 ± 0.70 ^a^	60.04 ± 0.30 ^a^	57.74 ± 0.73 ^ab^	55.14 ± 0.29 ^bc^	52.99 ± 0.87 ^c^	48.59 ± 0.50 ^d^	47.25 ± 0.45 ^d^	<0.01
	*p* value	1.000	0.299	0.316	0.280	0.263	0.558	0.717	0.184	0.177	
**Trypsin digestion**	OWP	67.59 ± 0.28 ^e^	70.54 ± 0.21 ^d^	73.42 ± 0.42 ^b^	75.10 ± 0.06 ^a^	72.78 ± 0.40 ^bc^	71.77 ± 0.43 ^cd^	67.73 ± 0.17 ^e^	63.45 ± 0.23 ^f^	60.49 ± 0.32 ^g^	<0.01
	VP	67.59 ± 0.28 ^d^	72.58 ± 0.28 ^bc^	74.11 ± 0.39 ^ab^	75.56 ± 0.54 ^a^	73.99 ± 0.31 ^ab^	71.66 ± 0.43 ^c^	67.07 ± 0.43 ^d^	64.87 ± 0.81 ^e^	61.17 ± 0.32 ^f^	<0.01
	*p* value	1.000	0.014	0.042	0.586	0.165	0.909	0.366	0.298	0.348	
**Total digestion**	OWP	74.92 ± 0.05 ^e^	81.17 ± 0.44 ^c^	83.94 ± 0.23 ^b^	87.89 ± 0.65 ^a^	83.68 ± 0.18 ^b^	77.66 ± 0.46 ^d^	73.91 ± 0.75 ^e^	71.14 ± 0.37 ^f^	67.28 ± 0.45 ^g^	<0.01
	VP	74.92 ± 0.05 ^g^	75.51 ± 0.19 f^g^	81.10 ± 0.46 ^bc^	87.69 ± 0.29 ^a^	81.98 ± 0.40 ^b^	78.49 ± 0.31 ^de^	77.70 ± 0.65 ^e^	79.74 ± 0.45 ^cd^	77.00 ± 0.24 e^f^	<0.01
	*p* value	1.000	0.001	0.018	0.853	0.051	0.344	0.055	<0.01	<0.01	

Note: ^a–g^ Mean values in the same packaging system during different frozen storage periods from the same index indicate statistically significant difference (*p* < 0.05).

## Data Availability

The data generated from the study are clearly presented and discussed in the manuscript.
